# Decrease in (Major) Amputations in Diabetics: A Secondary Data Analysis by AOK Rheinland/Hamburg

**DOI:** 10.1155/2016/6247045

**Published:** 2016-01-05

**Authors:** Melanie May, Sebastian Hahn, Claudia Tonn, Gerald Engels, Dirk Hochlenert

**Affiliations:** ^1^AOK Rheinland/Hamburg, Die Gesundheitskasse, Unternehmensbereich Ambulante Versorgung, Geschäftsbereich Selektivverträge, Kasernenstrasse 61, 40213 Düsseldorf, Germany; ^2^AOK Rheinland/Hamburg, Die Gesundheitskasse, Unternehmensbereich M-RSA/Finanzen/Controlling, Geschäftsbereich Controlling, Kasernenstrasse 61, 40213 Düsseldorf, Germany; ^3^Chirurgische Praxis am Bayenthalgürtel, Bayenthalgürtel 45, 50968 Köln, Germany; ^4^Ltd. Arzt Abteilung, Wundchirurgie St. Vinzenz Hospital Köln, Merheimer Strasse 221, 50733 Köln, Germany; ^5^Centrum für Diabetologie, Endoskopie und Wundheilung, Merheimer Strasse 217, 50733 Köln, Germany

## Abstract

*Aim*. In two German regions with 11.1 million inhabitants, 6 networks for specialized treatment of DFS were implemented until 2008. Data provided for accounting purposes was analysed in order to determine changes in the rate of diabetics requiring amputations in the years before and after the implementation.* Method*. Data covering 2.9 million people insured by the largest insurance company between 2007 and 2013 was analysed by the use of log-linear Poisson regression adjusted for age, gender and region.* Results*. The rate of diabetics needing major amputations fell significantly by 9.5% per year (*p* < 0.0001) from 217 to 126 of 100,000 patients per year. The rate of diabetics needing amputations of any kind fell from 504 to 419 of 100,000 patients per year (*p* = 0.0038).* Discussion*. The networks integrate health care providers in an organised system of shared care. They educate members of the medical community and the general public. At the same time, a more general disease management program for people with diabetes was implemented, which may also have contributed to this decrease. At the end of the observation period, the rate of diabetics requiring amputations was still high. For this reason, further expansion of organised specialized care is urgently needed.

## 1. Introduction

Diabetic foot syndrome (DFS) is a lifelong consequence of diabetes mellitus which occurs in active and inactive phases. It may place the mobility of those affected under threat and consequently their independence, quality of life, and ability to work. In some cases, it may be fatal. In particular, after amputations above the ankle, mobility is impaired, as half of those affected are no longer able to walk independently [1, 2]. An important aim in the care of people with active DFS is therefore to avoid these so-called major amputations. According to the data currently available, 5–10% of people with active DFS are affected by this in standard care, while the figure in specialised care is 2–3.5% [3–5].

A substantial proportion of the spending on diabetes care in Germany is attributable to the DFS [6, 7]. Major amputations in particular, with their follow-up costs, entail significant levels of spending [8]. The development of care for people with DFS receives high priority all over the world [9].

Delivery of care to the patient is unavoidably of an interdisciplinary and interprofessional nature. It requires coordinated cooperation between all of the parties involved, which in the regions of Rhineland (9.4 million inhabitants) and Hamburg (1.7 million) joint forces into six separate regional networks since 2002. These networks integrate hospital departments, doctors and nurses working in the outpatient field, and orthopaedic shoemakers and podiatrists as healthcare service providers working in independent facilities. Coordinated treatment paths, regular quality circles, visiting physician programmes, and open benchmarking are some of the methods used in shared patient care.

Within the German health care system, baseline medical care is covered by contracts between insurance companies and organised physicians. They are called “collective contracts” because all adequately specialized physicians are free to participate. These contracts are highly regulated by federal and regional law. Disease management programs are among these collective contracts. Additionally, insurance companies can conclude contracts with groups of health care providers to offer extra services to their customers. For these contracts called “selective contracts,” insurance companies are allowed to select the participating providers. AOK Rhineland/Hamburg (AOK RH) is a prime insurance company in these regions and aims to improve the care given to people with diabetes. To achieve this, in addition to a disease management program (DMP Diabetes), AOK RH together with other insurance companies supported the development of networks for the treatment of people with a diabetic foot syndrome since 2005 through selective contracts (DFS SC). After a trial period from 2005 to 2008, these contracts were concluded with network participants throughout the entire area covered by the AOK RH. From the very beginning of the contract, the aim was to produce an effect on the region as a whole. Therefore, the networks began at an early stage to make offers of further education to other facilities within the contract region. To this same aim, second-opinion procedures prior to major amputations were made available and awareness campaigns were conducted.

Publications on the incidence of amputations in Germany have so far been limited to the analysis of hospital stays with amputation events without reference to individuals. It has been argued that a change of strategy from partial amputations performed consecutively towards a unique procedure could result in reduced amputation figures in spite of an increase in the number of individuals affected and therefore a distortion of the perception of the result. The present work identifies not only the hospital stays with amputations performed, but also the number of people affected.

## 2. Materials and Methods

We analysed accounting data for the years 2007 to 2013 of the AOK RH in accordance with Sections 295, 300, and 301 of Social Security Code Book Five. This data contains information on the diagnoses according to the International Classification of Disease (ICD-10 GM), drug prescriptions according to the Anatomical Therapeutic Chemical- (ATC-) code, and surgical procedures according to the German Procedure Classification (OPS). The diagnosis of diabetes was considered to have been confirmed if indicated by more than one statement independently. These were similar to other investigations from the German healthcare system [10–12]:

(i) A 3-digit diagnosis (ICD E10^*∗*^ to E14^*∗*^) in at least 3 of 4 consecutive quarters at the level “certain” according to the ICD 10 GM.
(ii) At least two prescriptions of antidiabetic agents (ATC A10) within 12 months.
(iii) A prescription of antidiabetic agents and a diabetes diagnosis or a glucose or HbA1c measurement within 12 months.

Major amputations were considered to be those performed at the level of the ankle or above (OPS 5-864, 5-869.0), whereas minor amputations were amputations below the ankle (OPS 5-865), which is also analogous to earlier investigations [13, 14].

Absolute frequencies were normalised to 100,000 diabetics. Adjusted amputation frequencies were presented by means of regression analysis.

The figures were compiled separately for each of the 27 regions in Rhineland and Hamburg and presented together. The breakdown corresponds to the administrative structures of AOK RH and takes into account regional specificities.

### 2.1. Statistics: Poisson Regression

For each of the 27 regions in Rhineland and Hamburg, the number of diabetics and their gender and age distribution, as well as the amputations themselves were determined for each year during the period investigated. This took into account possible changes in demographic developments resulting from changes in age and gender distribution or the number of insured individuals.

Using a log-linear Poisson regression [15], the annual frequency of amputations within each region was modelled with adjustments according to age and gender. We additionally applied two different offset variables: in Model 1, the offset was the absolute number of diabetics in the year under consideration within each region; in Model 2, it was the number of diabetics in the year 2007 held constant over all years. The second modelling procedure was added in order to eliminate the possible effect of any change in coding behaviour over the years.

The Poisson regression was performed using SAS version 9.2 of PROC GENMOD.

## 3. Results

### 3.1. General

**Figure 1:**
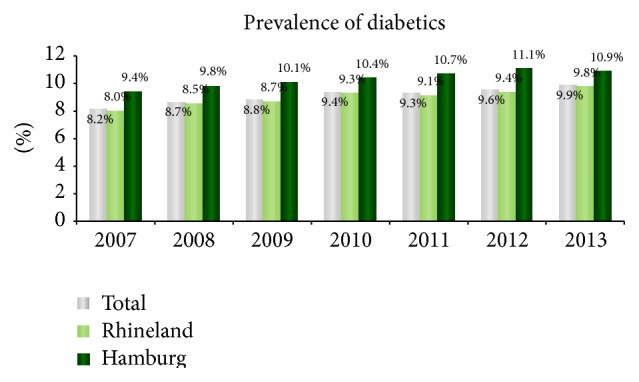
Prevalence of diabetics covered by AOK RH, 2007–2013.

Among approximately 2.9 million individuals insured by AOK RH in 2007, the diabetes prevalence was 8.2%. This figure rose in 2013 to 9.9%, with levels being 10.9% in Hamburg and 9.8% in Rhineland, respectively (Figure 1). The proportion of women was 37.2% overall and the average patient age was 69.3 (±13.8). The total number of hospital stays with amputations carried out on 6,958 diabetics in the period from 2007 to 2013 was 11,436 (3,607 with major and 7,829 with minor amputations).

### 3.2. Structured Care

**Figure 2:**
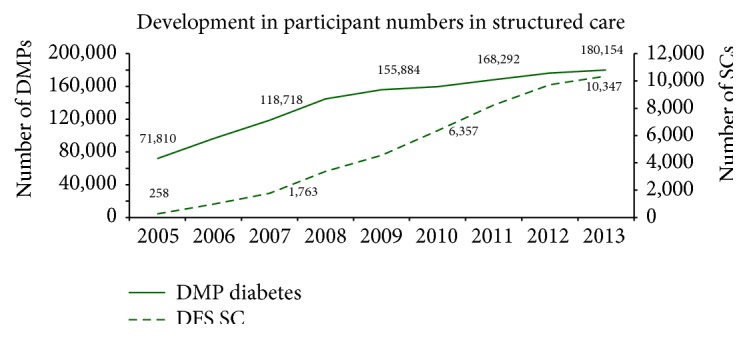
Development in participant numbers in the DMP and DFS selective contract (SC).

In 2013, over 10,000 people with diabetes and DFS received structured care in networks in accordance to contracts provided by the AOK RH. In addition to the increasing numbers of participants in structured care, the proportion of diabetics cared for in the Disease Management Program (DMP) also rose to around 65% and therefore amounted to over 180,000 individuals in 2013 in absolute terms (Figure 2). Of all policyholders with diabetes who underwent amputations, 777 (11.2%) were cared for in networks (SC), with 22.4% of these undergoing major amputations (Table 1).

### 3.3. Amputations

**Figure 3:**
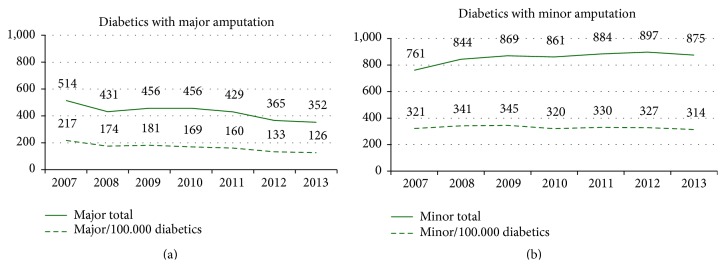
Number of diabetics with major or minor amputation, 2007–2013.

It was shown that over the course of seven years up to 2013, there was a significant reduction of 41.7% in the number of patients undergoing a major amputation (*p* < 0.0001). The proportion of people with minor amputations fell by 2.1% (*p* = 0.6624) (Table 2, Figures 3(a) and 3(b)). The proportion of those who required any form of amputation fell by 17.0% (*p* < 0.0001) (results normalised in each case to 100,000 diabetics). In total, 1,537 (22.1%) diabetics underwent multiple amputations over the years and can therefore be assigned to more than one year.

The results of the Poisson regression did not provide any indications of over- or underdispersion (deviance/*df* = 1.35 (Model 1) and 1.10 (Model 2)).

When adjusted for age and gender distribution for each region, a significant reduction in the number of individuals undergoing major amputations of 9.5% (*p* < 0.0001) is found across all of the years. If it is assumed that the number of diabetics remains constant (Model 2), an annual decline of 8.50% (*p* = 0.0002) is recorded. The number of people affected by amputations, regardless of whether these were major or minor, fell annually by 3.7% (*p* = 0.0038).

## 4. Discussion

The incidence of major amputations varies worldwide between 56 and 6,000 for every 100,000 people with diabetes [16]. This variability is caused not only by differences in health care, but also by uncertainties regarding the diagnosis of the diabetic disease and whether all of the amputation events performed are recorded [17]. The incidence also varies within countries. For example, at 151 Primary Care Trusts (PCTs) in England between 2007 and 2010, the figures varied from 64 to 525 per 100,000 [18]. In Ipswich (UK), the introduction of specialised care which completely replaced the previous form of care observed a reduction in major amputations in the years 1995 to 2005 from 364 to 67 per 100,000 people with diabetes [19]. In the study presented here, the number of those affected fell from 217 to 126 per 100,000 diabetics. The number of hospital stays with an event decreased from 263 to 146 per 100,000 diabetics. This need for improvement which still exists in the international comparison might be attributable to specific aspects of the German healthcare system. Generally speaking, all hospitals with their own surgical departments can charge fees for major amputations. The complete replacement of the existing form delivering care by an alternative form is not possible here.

The specialised care is provided as an additional offer to the standard care. Indeed, only a minority of the amputations examined here were performed on patients who received care in the networks of the selective contract. The fact that the care would only be partially taken over by the networks was already foreseeable when the intervention was planned; therefore, the introduction was accompanied by a number of measures such as advanced training courses, offers of second opinions, and awareness campaigns in order to achieve a broad effect.

Two previous population-based studies of the care for people with DFS in Leverkusen, a city within the area studied here, showed a decrease in the number of amputations over the entire period under investigation (1990–2005) [20] which had not yet been seen in the years 1990–1998 [21]. In this survey, the reduction was attributed to a change in the type of care, which also formed part of the development of the regional networks.

Previous evaluations of amputation incidence from accounting data [22–24] were case and not individual related. Therefore, it was not possible to state how many people with diabetes underwent amputations and the extent to which the development in the absolute surgical figures affected the number of patients involved. This is illustrated by the study presented here, which covers the insured from two major regions and uses the figures of the largest health insurance company in these regions.

Furthermore, the analysis of the number of patients affected shows for the first time that in Germany there has been a significant decrease in the number of people with diabetes who require amputations. The number of people affected by minor amputations is falling only by a lesser extent, which is partly attributable to the fact that minor amputations are being carried out instead of major amputations.

The limitations of the study relate in particular to the selection of the patients affected due to their membership of AOK RH and the development in the incidence of the diabetes diagnoses documented. However, the selection bias remained constant over the observed period; since there were no mergers of AOK RH with other health insurance companies, no other trends became apparent among the insured and the number of insured individuals remained more or less the same. The increase in the number of diagnosed diabetes cases might have been attributable in part to a change in coding behaviour. For this reason, a second modelling procedure was carried out which assumed that the prevalence of diabetes remained unchanged. In this evaluation, there was also a very clear and significant reduction in the incidence of insured individuals undergoing amputations in particular major amputations.

## 5. Summary Assessment

The figures presented, which are based on routine data, confirm a very significant improvement in the care of people with DFS. The number of people affected who underwent major amputations with their serious consequences fell dramatically during the seven years after the introduction of organised specialised care. Both the structured treatment program DMP Diabetes and the specialized “DFS” contract are used by a large proportion of affected people in Rhineland and in Hamburg. However, the majority of those who underwent an amputation event did not use specialized care offered by the contract. The increasing number of patients in this contract alongside with the efforts to induce advances in the nonspecialized standard care might explain that improvement and should be investigated in further studies. For this reason, the expansion of the care of people with diabetic foot syndrome in structured foot networks is indispensable in the future.

## Figures and Tables

**Table 1:** Overview of selective contract (SC) participants and amputation frequency.

People with diabetes and amputation from 2007 to 2013	6,958
SC participants with amputation (major or minor)	777
SC participants with major amputation	174
SC participants with minor amputation	613
Proportion of SC participants undergoing major amputations compared to the total number of amputations	22.4%

**Table 2:** Absolute number of hospital stays with amputation events and diabetics, proportion of diabetics with amputation compared to total number of diabetics.

Year	Number of the insured	Number of insured diabetics	Number of hospital stays with amputations			Number of diabetics with amputation			Number of diabetics with amputation/100,000 diabetics		
			Major	Minor	Amputation (major + minor)	Major	Minor	Amputation^*∗*^	Major	Minor	Amputation^*∗*^
2007	2,908,300	237,164	619	975	1,594	514	761	1,196	217	321	504
2008	2,857,963	247,690	511	1,083	1,594	431	844	1,190	174	341	480
2009	2,850,008	251,623	559	1,120	1,679	456	869	1,253	181	345	498
2010	2,863,114	269,432	566	1,130	1,696	456	861	1,243	169	320	461
2011	2,881,479	267,708	515	1,120	1,635	429	884	1,245	160	330	465
2012	2,867,117	274,092	421	1,198	1,619	365	897	1,201	133	327	438
2013	2,817,703	278,647	416	1,163	1,579	352	875	1,167	126	314	419

^*∗*^Patients were counted only once in the corresponding year irrespective of the type of amputation (major and/or minor).
